# Long-term outcome of patients after a single interruption of antiretroviral therapy: a cohort study

**DOI:** 10.1186/1756-0500-5-578

**Published:** 2012-10-24

**Authors:** Carmen Machado, María José Ríos-Villegas, Juan Gálvez-Acebal, Angel Domínguez-Castellano, Felipe Fernández-Cuenca, Virginia Palomo, Miguel Angel Muniain, Jesús Rodríguez-Baño

**Affiliations:** 1 Infectious Diseases Unit, Hospital Universitario Virgen Macarena. Seville, Avda Dr Fedriani 3, Seville, 41009, Spain; 2 Departament of Medicine, University of Seville, Seville, Spain

**Keywords:** HIV-1, Antiretroviral therapy, Treatment interruption, Outcome, Cohort study

## Abstract

**Background:**

To describe the long term outcome of patients who interrupted highly active antiretroviral therapy (HAART) once, identify the variables associated with earlier need to re-start HAART, and the response when therapy was resumed. A retrospective observational cohort of 66 adult patients with HIV-1 infection who interrupted HAART with a CD4+cell count ≥350 cells/μL and undetectable viral load (VL) was performed. The pre-established CD4+ cell count for restarting therapy was 300cells/μL. Cox regression was used to analyse the variables associated with earlier HAART reinitiation.

**Results:**

The median follow-up was 209 weeks (range, 64–395). Rates of HIV-related or possible HIV-related events were 0.37 (one case of acute retroviral syndrome) and 1.49 per 100 patient-years, respectively. Two patients died after re-starting therapy and having reached undetectable VL. Three patients suffered a sexually transmitted disease while off therapy. Fifty patients (76%) resumed therapy after a median of 97 weeks (range, 17–267). Age, a nadir of CD4+ <250 cells/μL, and a mean VL during interruption of >10,000 copies/ml were independent predictors for earlier re-start. The intention-to-treat success rate of the first HAART resumed regimen was 85.4%. There were no differences by regimen used, nor between regimens that were the same as or different from the one that had been interrupted.

**Conclusions:**

Our data suggest highly active antiretroviral therapy may be interrupted in selected patients because in these patients, when the HAART is restarted, the viral and clinical response may be achieved.

## Background

High active antiretroviral therapy (HAART) has completely changed the prognosis of HIV infection [1]. Although nowadays HAART is recommended as a life-long therapy [2-4], interruptions of therapy in patients with undetectable HIV viral load (VL) while on therapy have been evaluated, both as fixed periods with and without therapy (structured interruptions) and as a strategy in which therapy is re-started after interruption when the CD4+ lymphocyte count diminish below a predetermined level (CD4+ guided interruptions). A meta-analysis concluded that structured interruptions had not demonstrated short-term safety; and long term data were lacking for CD4+ guided interruptions [5]. Results from a later meta-analysis suggested that interruption strategies are associated with a low risk of death or aids-defining events, although the risk is not significant if the threshold for restarting HAART is established at a relatively high level of CD4+ cell count [6].

Beyond pre-established strategies, interruption of HAART are more frequent that desired due to different reasons [7], such as patient’s decision to stop therapy, reluctance to alternatives in the event of intolerance to the current regimen, etc. When an interruption occurs, questions about the short and long term risk of complications, and the probability of reaching virologic and immunologic control once therapy is re-started frequently arouse.

The objectives of this study were to describe the very long term outcome of patients who interrupted HAART for ≥3 months while being in good immunologic condition and with undetectable VL, to evaluate the variables associated with an earlier need to re-start therapy, and to analyse the virologic and immune response to the reintroduction of HAART.

## Patients and methods

### Study design

A retrospective cohort analysis was performed. Patients were considered eligible if included in Hospital Universitario Virgen Macarena cohort of adult patients (age >18 years) patients with chronic HIV-1 infection, and had interrupted the antiretroviral drugs for at least 12 weeks between January 2001 and December 2004. Only patients who interrupted a stable HAART regimen while clinically stable, with undetectable VL (<50 copies/mL) and stable CD4+ cell count ≥350/μl for the last 6 months were included.

Patients were visited at least every 12 weeks. In all routine visits, a clinical examination and routine blood tests including a CD4+ cell count and RNA HIV-1 viral load testing (Amplicor HIV-1 Monitor Test version 1.5, Roche Diagnostic System) were performed. Decisions about restarting HAART were discussed in every visit with the patients; the following criteria were pre-established as indications to reinitiate therapy: CD4+ cell count < 300 cells/μL (the attending physicians had previously agreed to consider this CD4+ count threshold for resuming therapy), pregnancy, acute retroviral syndrome, thrombocytopenia, and any other B or C event [8]. Data were collected from the structured charts until death or lost to follow-up, and patients were censored in October 2008 to perform this analysis.

The study was approved by the Ethic Committee from Hospital Universitario Virgen Macarena. The need to obtain written consent informs was waived because of the retrospective nature of the study.

### Variables and definitions

From all patients, the following variables were collected: age, gender, ethnics, risk behaviours, number of years since the diagnosis of HIV infection, toxic habits, HCV and HBV coinfection, HIV category before interruption, CD4+ cell nadir and in the interruption, number and duration of HAART regimens, previous virologic failures, and previous available resistance-related mutations.

The main endpoints of the study were the occurrence of HIV-related or possibly related events and death from any cause. The following events were considered HIV-related: acute retroviral syndrome, all B events according to 1993 CDC classification [8], and all aids-defining events from the 2008 CDC classification [9]. The following were considered as possibly HIV-related: bacterial pneumonia, any type of malignancy, thrombocytopenia, and death from any cause except if clearly unrelated to HIV, its complications or its influence on other conditions. All other events were considered as non HIV-related.

Secondary end-points were: time until HAART was restarted and response to HAART after interruption. The latter was defined as failure in an intention to treat analysis if any of the following circumstances occurred: undetectable VL (<50 copies/ml) had not been reached in the visit when the HAART regimen was changed or at the end of follow-up [10], the HAART regimen had to be changed because of intolerance, death from any cause, or HIV-related events [8,9]. Otherwise it was considered successful. Virologic failure was also analysed per protocol in patients who did not change the HAART regimen because of intolerance. Adherence to therapy was measured by the SMAQ3 questionnaire [11].

### Statistical analysis

Time until reinitiation of HAART was studied using Kaplan-Meier curves. The crude association between exposure to the different variables and the time until restart of therapy was studied by the log-rank test. Multivariate analysis was peformed by Cox regression. Analysis were performed with SPSS 15.0.

## Results

We included 66 patients. Their features are summarised in Table 1. The reasons for treatment interruption included: proposal of the attending physician, patient´s ance and poor adherence (Table 1). The median follow-up from HAART interruption was 209 weeks (range, 64–395), with a total of 287 patients-year. Over the study period, 3 patients (4.54%; 1.04 events per 100 patients-year) developed HIV-related events (an acute retroviral syndrome and two thrombocytopenias during HAART interruption); no patient developed any C event. Three patients (4,54%; 1.39 events per 100 patients-year) developed 4 events possibly related to HIV (Table 2); 2 of them occurred during HAART interruption (2 bacterial pneumonia) and other 2 after HAART was restarted, in both cases when undetectable VL has been reached (decompensation of liver cirrhosis due to HCV 23 weeks after restart; and Hodgkin disease, 100 weeks after restart; both patients died as a consequence of the events; both patients had stopped HAART on their own). Other non HIV-related events (all happened during interruption) were: 2 acute hepatitis due to VHA (probably sexually transmitted), one urethritis due to *Neisseria gonorrhoeae*, one pyelonephritis due to *Escherichia coli*, and one hypertensive crisis. There were no other cardiovascular events. If all potentially HIV-related events were counted including those occurring after HAART was resumed, the rate of events would be 1.64 events per 100 patients-year (follow up included the time after therapy resume).

**Table 1:** Features of included patients. VL: viral load. HAART: highly active antiretroviral therapy

**Variable**	**Number of patients (percentage), except where specified**
Median age (range)	37 years (23–63)
Male gender	45 (68)
Risk category	
Injection drug users	28(42)
Men who have sex with men	16 (24)
Heterosexual	22 (34)
Toxic habits	
Current smoking	46 (69)
Alcholism	22 (33)
Methadone Maintenance Treatment	20 (30)
Median time since diagnosis of HIV infection (range)	10 years (2–16)
CDC category	
A	54 (82)
B	6 (9)
C	6 (9)
Number of previous antiretroviral regimens	
1	13 (20)
2	16 (24)
3 or more	37 (56)
Median time on HAART before interruption (range)	284 weeks (60–512)
Adherence <80% to any previous antiretroviral therapy	20 (30)
Previous virologic failure with any antiretroviral regimen	
No	53 (81)
Yes	13 (19)
HCV coinfection	33 (50)
HBV coinfection	2 (3)
Median HIV-1 VL at diagnosis (range)	23.750 copies/mL (100–1.511.000)
Median nadir CD4+ cell count (range)	252 cells/μL (50–552)
Main reason for the interruption of HAART	
Proposed by physician	33 (50)
Intolerance to current regimen	20 (30,3)
Decision of the patient	10 (15,2)
Problems for an appropriate adherence	3 (4,5)
Median CD4+ cell count at interruption (range)	745 (350–2132)

**Table 2:** Events detected during the follow up of 66 patients who interrupted antiretroviral therapy

**Patient**	**Event**	**Weeks after interruption**	**Situation when event occurred**	**CD4+ cell count in the interruption**	**CD4+ cell count when the event occurred**	**Outcome**
1	Bacterial pneumonia	48	Interrupted	480	234	Cured
	Hodgkin’s lymphoma	164	100 weeks after HAART was restarted (VL<50 since week 4)	480	415	Dead
2	Decompensation of HCV-related liver cirrhosis	97	23 weeks after HAART was restarted (VL<50 since week 4)	415	120	Dead
3	Thrombocytopenia	196	Interrupted	366	234	Resolved after HAART restarted
	Bacterial pneumonia	200	Interrupted	366	234	Cured
4	Thrombocytopenia	161	Interrupted	1089	580	Resolved after HAART restarted
5	Acute retroviral syndrome	12	Interrupted	576	415	Resolved after HAART restarted

**Figure 1:**
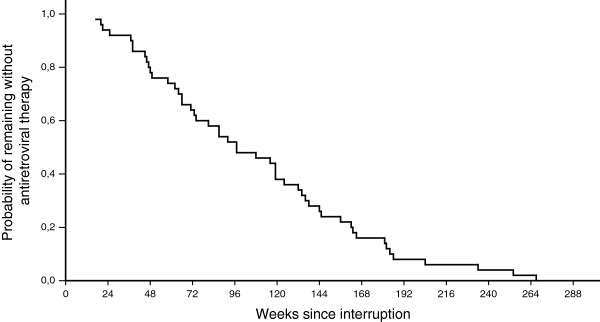
Probability of remaining off antiretroviral therapy after interruption.

**Figure 2:**
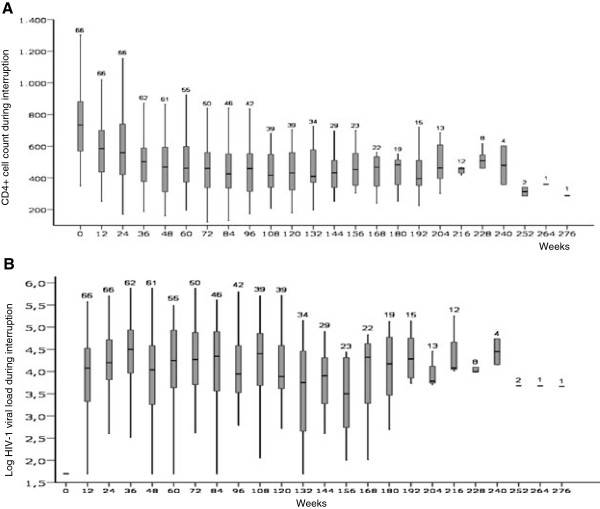
**Evolution of CD4+ cell count (2A) and HIV-1 viral load (2B) after HAART interruption.** Data are expressed as median values (horizontal lines), interquartile range (grey boxes), and percentile 95 (vertical lines). The number of patients with data in each time period is provided over each vertical line.

At the end of the study period, 50 patients (76%) had restarted HAART. The median duration of interruption was 97 weeks (range, 17–267). The probability of remaining without HAART is shown in Figure 1. The evolution of CD4+ cell count and VL during the interruption is shown in Figure 2. A nadir CD4+ cell count <250 per μl (p<0.001), and category B or C (p=0.003) were associated with shorter period of interruption in the crude analysis; these variables were included in the multivariate analysis together with age, and mean VL during interruption >10.000 copies/ml (since the p value for the latter was <0.1). By a backward process, the variables selected as predictors of shorter time of interruption were: higher age (HR per year = 1.07; 95% CI: 1.02-1.12; p=0.002), a nadir CD4+ cell count <250 per μl (HR = 3.70; 95% CI: 1.92-7.14; p <0.001) and mean VL during interruption >10.000 copies/ml (HR = 3.51; 95% CI: 1.56-7.14; p=0.002). The remaining variables were not associated with shorter period of interruption in the crude analysis either with the time from resuming therapy to reach undetectable load.

**Figure 3:**
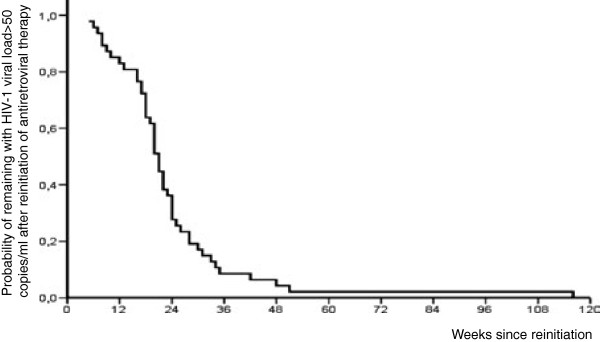
Probability of remaining with detectable HIV-1 viral load (>50 copies/mL) after reinitiating antiretroviral therapy.

In the analysis of response to restarted HAART, we excluded 2 patients whose follow up at the end of study period was <12 weeks; thus, 48 patients were evaluated. Among them, the median follow-up after HAART restarted was 101 weeks (range, 12–254). The median CD4+ cell count when HAART was restarted was 266 (range, 40–999), and the median VL was 51.400 copies/ml (range, 50->100.000). The probability of remaining with detectable HIV-1 viral load (>50 copies/ml) after reinitiating antiretroviral therapy is shown in Figure 3.

The main reason for restarting HAART was the decrease in the CD4+ cell count in 43 patients (88%), HIV-related or possibly related events in 3 (6%), patient’s request in 2 (4%), and pregnancy in 1 (2%). As explained above, there were no B o C events after HAART was restarted, and 2 patients died (Table 2).

All patients received 2 nucleoside or nucleotide reverse transcriptase inhibitors (NRTI) plus another drug: a non-nucleoside reverse transcriptase inhibitor (NNRTI), 25 patients; a boosted protease inhibitor (PI), 17 patients; or another drug, 4 patients. In an ITT analysis, the success rate were 84%, 88.2% and 100%; the differences between groups were not statistically significant (p value for NNRTI vs PI, 0.7).

The initial regimen was based on the same groups of drugs than the one that was interrupted in 25 patients (52%). Among them, 16 received an initial regimen based on NNRTI; 14 reached undetectable VL, one was changed to PI because of intolerance, and the other was changed to PI because of virologic failure (a genotypic study could not be performed because the VL was always <500 copies/ml). Eight received an initial regimen based on PI; 7 reached undetectable VL and the other was changed because of intolerance. The other patient was treated with 3 NRTI and reached undetectable VL.

The initial regimen was based on a different group of drugs than the one that was interrupted in 23 patients. Among them, the initial regimen after interruption was based in a NNRTI in 9 patients; 7 of them reached undetectable VL, one was changed because of intolerance, and the other needed to be changed to a PI-based regimen because of virologic failure; genotypic test could not be performed. Nine received an initial regimen based on PI; 8 reached undetectable VL, and one failed; mutations found were 62V, 75I, 118I, and 184I (reverse transcriptase gene) and 77I (protease gene). This patient had a low adherence and was still in virologic failure at the end of follow-up. The 4 patients with regimens base on other drugs reached undetectable VL.

In an ITT analysis, we found no difference between patients who restarted HAART with a regimen based on the same families of drugs that had been interrupted and those who restarted with a different regimen (success rate, 88% vs 91%, p=0.7).

**Figure 4:**
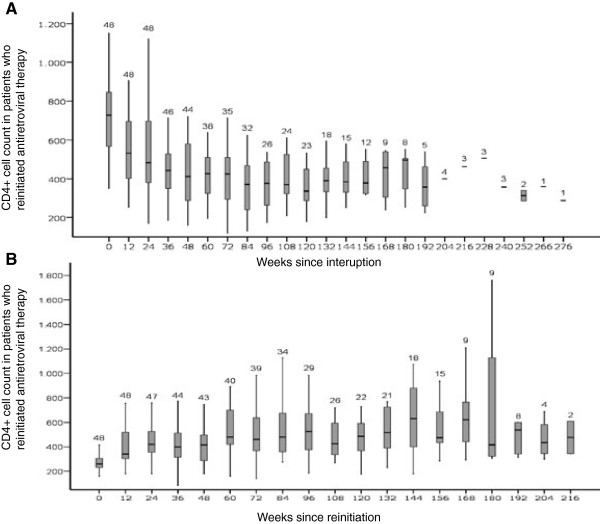
**Evolution of CD4+ cell count during HAART interruption (4A) and after HAART restart (4B) in patients who resumed antiretroviral therapy.** Data are expressed as median values (horizontal lines), interquartile range (grey boxes), and percentile 95 (vertical lines). The number of patients with data in each time period is provided over each vertical line.

The evolution of CD4+ cells is shown in Figure 4. In patients with 96 week follow up after resuming the therapy, the median CD4+ cell count was 727 cells/ml when HAART was interrupted, and 529 cells/ml at 96 week after HAART was restarted (p=0,001).

## Discussion

Our study show that long term prognosis of patients who interrupted HAART once for more than 12 weeks, while being in a stable situation, and with the intention to restart HAART once CD4+ cells were ≤300 per μL was good, although some considerations need to be done.

The hypothesis for investigating HAART interruptions as a strategy included: the potential effect of viral rebound in stimulating the immunologic response against HIV, and the potential reduction in HAART-related adverse events, in the fatigue of patients, and in the costs of therapy. Structured treatment interruptions, with fixed cycles on and off therapy, have not demonstrated a comparable short-term safety in comparison with continuous treatment [5]. On the other side, CD4+−guided interruptions have been investigated both in randomised clinical trials and cohort studies. A recently published meta-analysis considered 7 randomised clinical trials (although only the 4 of them with >100 patients-year of follow-up were actually included), and 11 cohort studie [6]. Even though the heterogeneity of the studies and the possibility of a publication bias limit the conclusions of the meta-analysis, the results suggest that the risk of events (including aids-defining events and deaths) were higher in patients with interruptions; however, the effect was small (one death per 100 patients-year). In the cohort studies, the rate of events ranged between 0 (8 studies) and 3.2 per 100 patients-year, and was higher when HAART was restarted with <250 CD4+cells per μl. Thus, structured interruptions of HAART are not recommended out of well-controlled research studies [10]. However, a recent study suggested that CD4+ cell-guided treatment interruptions may be safe [12]. To our knowledge, our study includes the more prolonged follow-up, and we could not detect any aids-defining event; the rate of HIV-related events is intermediate among the published cohorts. However, it should be noted that our patients only interrupted HAART once.

Two patients in our cohort died. Both had interrupted HAART on their own, and both reached undetectable VL after restarting therapy. However, one developed a Hodgkin’s lymphoma, and the other had a decompensation of HCV-related liver cirrhosis. Since non-aids-defining malignancies and progression of HCV-related liver fibrosis are conditions in which HAART may have a preventive role (though the issue is controversial), this should be considered in the case of an interruption. Also, the fact that the CD4+ cell count level at the interruption was not reached again after therapy was resumed, is another aspect to be considered.

The interruptions analysed in our study occurred in a specific context in the history of HAART: the previous hypothesis that HIV infection could be cured was proved false; the late side effects of some drugs were emerging; and the alternative regimens were scarce. Obviously, the context today is different. However, based on the long term follow-up of our series, and since still most present regimens are based on 2 NRTI plus a NNRTI or PI, we think our data may be useful in the decision-making process and advice to patients when an interruption of HAART has happened or is being discussed. The variables associated to a shorter time for restarting HAART (higher age, CD4+ nadir <250 cells/μL, and a higher VL during the interruption) are sound and in agreement with previous investigations [13-16], and may be used to identify patients who would need a more close follow-up.

A key question in patients who interrupt HAART is the possibility of HIV transmission [17]. In our series, and even though this aspect had been specifically discussed with all of them, 3 of our patients suffered a sexually transmitted disease, indicating that they may have also transmitted HIV during the same risk practise. There is no doubt that this is a very important issue to consider and discuss in the cases of interruptions.

One potential worry after an interruption is whether the probability of reaching again virologic control is compromised. The vast majority of our patients succeeded in controlling VIH replication, although undetectable VL was reached somehow late in many patients. Even though the number of patients is low, we did not find differences between NNRTI- and PI-based regimens, or if the regimen was similar to the one that was interrupted or not. Interruptions of NNRTI-based regimens has been associated to selection of mutations conferring resistant to these drugs in previous studies, although this may be more frequent in the case of multiple interruption [18,19] In our study, only one patient among the 16 who interrupted and restarted a NNRTI-based regimen showed a virologic failure (mutations cannot be studied).

Our study has several limitations that should be considered. First, it was performed in only one center. Second, metabolic parameters, inflammation markers or cardiovascular risks were not assessed, therefore potential implications of the HAART interruption related to these items could not be analysed. And third, we could not compare the data with a control group of matched patients who continued therapy.

## Conclusions

Our data suggest that restarting HAART when a single interruption occurs is not an emergency and that it is safe in most patients to wait while reinforcing the important of a correct adherence and the alternatives to potential adverse effects of HAART. The risk of non-aids-defining events, worse evolution of coinfections, and transmission of the HIV should be specifically discussed.

## Competing interests

Two authors of this article, Carmen Machado and Virginia Palomo were recipients of grants from Andalusian Public Foundation for the management of Research in Health of Seville (FISEVI). Five authors, J. Galvez, A. Domínguez, F. Fernández-Cuenca, M A. Muniain and J. Rodríguez Baño received funding for research from Ministry of Science and Innovation, Institute Carlos III of Health - co-financed by European Development Regional Fund "A way to achieve Europe" ERDF, Spanish Network for the Research in Infectious Diseases (REIPI RD06/0008). Anyway, the sources of funding have not had an involvement in the design of the study, the collection of data, the analysis or interpretation of these, in the drafting of the manuscript neither in the decision to send it for publication.

## Authors’ contributions

FFC carried out the RNA HIV-1 viral load test and resistance testing. CM and VP participated in the data recollection from clinical histories and the inclusion in database MJRV, JGA, ADC, MAM and JRB selected the patients, attended the routine visits, the clinical examination and advised them of any possible higher risk of HIV transmission to sexual partners during the interruption . JRB, MAM and CM participated in the design of the study and performed the statistical analysis. JRB and MJR conceived of the study, and participated in its design and coordination. All authors helped to draft the manuscript, read and approved the final manuscript.
